# High Resolution Imaging and Fixation Analysis of Eccentric Preferred Retinal Loci in Macular Diseases

**DOI:** 10.1167/iovs.66.5.18

**Published:** 2025-05-08

**Authors:** Olubayo U. Kolawole, Ethan Bensinger, Jessica Wong, Nicholas Rinella, Katharina G. Foote, Hao Zhou, Ruikang K. Wang, Jacque L. Duncan, Austin Roorda

**Affiliations:** 1 Wayne and Gladys Valley Center for Vision, Department of Ophthalmology, University of California San Francisco, San Francisco, California, United States; 2 Herbert Wertheim School of Optometry and Vision Science, University of California Berkeley, Berkeley, California, United States; 3 Department of Bioengineering, University of Washington, Seattle, Washington, United States; 4 Department of Ophthalmology, University of Washington, Seattle, Washington, United States

**Keywords:** preferred retinal loci (PRL), macular diseases, adaptive optics scanning laser ophthalmoscopy (AOSLO), microperimetry, fixational stability

## Abstract

**Purpose:**

The purpose of this study was to characterize the preferred retinal locus (PRL) structure and fixational eye movements in eyes with macular atrophy.

**Methods:**

Four participants (1 each with macular atrophy due to congenital rubella, Best macular dystrophy, cuticular drusen with macular atrophy, and Stargardt disease) were studied using adaptive optics scanning light ophthalmoscopy (AOSLO), optical coherence tomography (OCT), OCT angiography (OCT-A), and microperimetry. Imaging sessions were repeated in three of the four participants. PRL and fixation stability were measured with AOSLO. Fixation stability was compared with healthy participants and participants with *RHO-* and *USH2A-*related retinitis pigmentosa (RP).

**Results:**

The PRL in participants with eccentric fixation was 0.44 to 1.92 degrees from the anatomic fovea and visual acuity was 20/40 or better. Cones at the PRL were not visible in confocal images, despite normal-appearing and more sensitive cones at greater eccentricities. OCT at the PRL showed intact external limiting membranes but hyporeflective and disrupted inner-segment outer-segment junctions. Fixation stability in participants with eccentric PRLs was no worse than participants with RP, all with foveal PRLs. The eccentric PRL group and the *USH2A* group with worse visual acuity (20/30 to 20/50) had fixation stabilities that were worse than the healthy controls.

**Conclusions:**

Participants adopt eccentric PRLs with hyporeflective cones and reduced sensitivity despite more sensitive and normal-appearing cones at greater eccentricities, suggesting that foveal proximity is prioritized over cone integrity in establishing a PRL. Fixation stability was similar among the four participants with eccentric fixation and those with RP, indicating that small shifts in the PRL from the anatomic fovea in our participants do not make fixation less stable.

The retinal region where images are placed during fixation is called the preferred retinal locus (PRL). In those without central vision loss, the PRL is generally at, or very near the anatomic fovea (retinal location with peak density of cones), but in the event of central vision loss resulting from macular atrophy or otherwise, humans develop new PRLs at new retinal locations.^1^ The PRL necessarily shifts due to a lack of sensory responsiveness from the original foveal region, and its location changes with progressive change in the atrophic lesion. Understanding the PRL is important for evaluating and planning rehabilitation strategies for participants with central vision loss.^2^

Numerous studies to characterize the PRL in eyes with central field loss have been published. Below, we summarize the general findings in the literature that are relevant to this study.

• The PRL is generally located outside the atrophic lesion and can be close to the visible edge of atrophy (more often the case in eyes with age-related macular degeneration [AMD]) or can be further away from the edge of the visible atrophic lesion, such as in Stargardt disease.^3^^–^^9^
• The PRL is located at anatomic locations that are most often superior, nasal, or temporal to the fovea, and is rarely located inferiorly.^3^^,^^6^^,^^8^^,^^10^ Note that choosing a PRL that is superior to the fovea in retinal coordinates places the scotoma above one's line of sight in the visual field.
• The PRL is not always in the location of best visual acuity or with the greatest retinal sensitivity.^4^^,^^6^^,^^8^
• Fixation stability, often quantified as the best fitting bivariate contour ellipse area (BCEA) encompassing a stated fraction of the fixation locations,^11^ is reported to be worse whenever an eccentric PRL is adopted. The BCEA was found to be as much as 25 times larger in eccentric fixators compared to healthy age-matched participants.^3^^,^^12^^,^^13^ Most studies report that fixation stability worsens with increased PRL eccentricity.^8^^,^^11^ Related, fixation stability worsens with the size of macular atrophy^2^ and the distance between PRL and the edge of the macular atrophy.^2^^,^^14^ However, reports of fixation stability and their magnitudes have been quite variable depending on individual differences, patient age, disease, time since central field loss, and difference in fixation stability analysis.
• Scanning laser ophthalmoscope images often show the PRL to be outside of the visible atrophic lesion. In some cases, however, the PRL appears to be inside a visible atrophic lesion indicating that there may be residual function, despite the appearance of photoreceptor loss.^15^^,^^16^
• Fundus autofluorescence (FAF) signals can indicate RPE absence (hypofluorescence) or RPE disease (hyperfluorescence and nonuniform FAF). Whereas PRLs are rarely chosen in hypofluorescent RPE regions, they are often chosen in areas with RPE disease in regions adjacent to the central scotoma.^6^^,^^8^
• Optical coherence tomography (OCT) imaging has revealed that PRLs can be chosen in regions with intact and visible external limiting membranes and intact RPE, but with disrupted or invisible inner segment/outer segment (IS/OS) junctions, and thinner outer nuclear layers.^8^^,^^17^^,^^18^

Despite extensive research, the anatomic and functional characteristics of the chosen PRL in eyes with central field loss are not fully understood, in part, because the conventional clinical tools used to evaluate the structure and function of the PRL do not have the ability to combine cellular level imaging with functional testing. Adaptive optics scanning light ophthalmoscopy (AOSLO) is a modality that can generate cellular-resolution images in the macula of living eyes^19^ and can identify the PRL with micrometer-scale accuracy.^10^^,^^20^^–^^22^ To date, the use of AOSLO to study the PRL and its underlying retinal structure and function in eyes with macular atrophy is limited to a single case report.^15^ In this study, we use a multi-modal, high-resolution imaging approach to characterize the PRL in participants with macular atrophy arising from four different conditions and compare fixation with participants who have retinitis pigmentosa (RP) with foveal fixation. Modalities include AOSLO, spectral-domain OCT (SD-OCT), swept-source OCT angiography (OCT-A), FAF, and fundus-guided microperimetry to understand more clearly the relationship between retinal structure and function at the PRL.

## Methods

### Study Participants

Approvals for this study and data collection were obtained from the Institutional Review Board of the University of California, San Francisco. Written informed consent was obtained from each study participant. Eyes with macular atrophy and visual acuity of 20/40 or better were selected for detailed study. We studied the better eye of each of the four participants, one with Best vitelliform macular dystrophy (participant 40146, left eye), one with congenital rubella (participant 40122, left eye), one with Stargardt disease associated with compound heterozygous pathogenic variants in the *ABCA4* gene (participant 30014, left eye), and one with macular atrophy associated with cuticular drusen (participant 40184, right eye). Twelve participants with RP, eight with *USH2A* mutations, and four with *RHO* mutations (previously reported in Ref. 23) with central fixation and visual acuity 20/50 or better were selected for comparison with our macular atrophy participants. Details for all participants are shown in the Table.

**Table.** Clinical Characteristics of Participants with Macular Atrophy and Retinitis Pigmentosa

Participant ID	Study Eye	Disease	Mutations and Protein Effect	BCVA at Latest Visit	Age	AOSLO-Derived Average BCEA, Degrees^2^)
40146	OS	Best vitelliform macular dystrophy	(*BEST1* c. 920 C>T; p. Thr307Ile)	20/32	57 y	0.054
40122	OS	Congenital rubella	None	20/32	56 y	0.167
30014	OS	Stargardt disease	*ABCA4* c.4457C>T, p.Pro1486Leu; c.672_703delinsN[6], p.Val225(?)fs*46	20/50	26 y	0.062
40184	OD	Cuticular drusen with macular atrophy	No disease-causing genes	20/40	48 y	0.079

40082	OS	*USH2A*-related ARRP	c.8522G>A, p.Trp2841* and c.11266G>A, p.Gly3756Ser	20/40	44 y	0.107
40151	OD	*USH2A*-related ARRP	c.11864G>A, p.Trp3955* and c.6835G>C, p.Asp2279His	20/40	58 y	0.083
40097	OS	*USH2A-*related Usher syndrome type 2	c.2299delG, p.Glu767SerfsX21 homozygous	20/32	34 y	0.061
40153	OS	*USH2A-*related ARRP	Deletion of exon 27 (c.5299-?_5572+?del) and c.9882C>G, p.Cys3294Trp	20/50	62 y	0.064

40039	OS	*USH2A-*related ARRP	c.2276G>T, p.Cys759Phe and c.2296T>C, p.Cys766Arg	20/16	48 y	0.058
40180	OD	*USH2A-*related ARRP	c.2299del, p.Glu767Serfs*21 and c.2276G>T, p.Cys759Phe	20/16	34 y	0.021
40110	OD	*USH2A-*related ARRP	c.11156G>A, p.Arg3719His and c.8659dup, p.Tyr2887Leufs*2	20/25	47 y	0.073
40163	OD	*USH2A-*related ARRP	c.2276G>T, p.Cys759Phe and c.1036A>C, p.Asn346His	20/16	49 y	0.096

30019	OD	*RHO-*related ADRP	c.152G>T p.Gly51Val	20/20	46 y	0.136
40167	OD	*RHO-*related ADRP	c.512C>G p.Ser270Arg	20/25	41 y	0.031
40095	OS	*RHO-*related ADRP	c.810C>A p.Ser270Arg	20/16	36 y	0.083
40183	OS	*RHO-*related ADRP	c.68C>A p.Pro23His	20/16	42 y	0.016

ADRP, autosomal dominant retinitis pigmentosa; ARRP, autosomal recessive retinitis pigmentosa; BCVA, best corrected visual acuity; OD, right eye; OS, left eye.

“None” indicates the participants did not undergo genetic testing.

### Genetic Analysis

Genetic testing was done using a next-generation sequencing retinal dystrophy panel with deletion/duplication analysis through the My Retina Tracker registry genetic testing study (NCT 02435940; 181 genes: 40146, 40184, 40039, 40151, 40110, and 40153; or 266 genes: 40180). All 50 exons of the *ABCA4* gene were sequenced on a research basis using the Illumina (San Diego, CA, USA) platform in participant 30014, as previously reported.^24^ Genetic testing was performed using an amplification refractory mutation system for the most common RP-causing variants in the *RHO* gene on a fee-for-service basis (Carver Nonprofit Genetic Testing Laboratory, Iowa City, IA, USA) in 30019; with a 67 gene retinal dystrophy next generation sequencing panel on a fee-for-service basis (EGL Genetics Laboratory, Tucker, GA, USA) in 40163; with a 13 gene autosomal recessive RP panel (Oregon Health & Sciences University, Portland, OR, USA) in 40082; with a 9 gene Usher syndrome panel in 40097 (GeneDx, Gaithersburg, MD, USA), analysis of the 5 coding exons and the flanking intronic regions of the *RHO* gene (40095 and 40183; University of Texas-Houston), or a 266 gene next generation sequencing panel (40167; Ocular Genomics Institute, Massachusetts Eye and Ear Infirmary, Boston, MA, USA) through the eyeGENE Research Consortium.^25^ Participants with autosomal recessive Stargardt disease associated with variants in *ABCA4*, and with autosomal recessive RP associated with likely pathogenic or pathogenic variants in the *USH2A* gene submitted samples from at least one first degree relative to confirm inheritance was in trans.

### Examination Procedures

Visual acuity was measured according to the Early Treatment of Diabetic Retinopathy Study (ETDRS) protocol.^26^ Visual acuity was performed first in an undilated state. Then, the pupils were dilated and fundus-guided microperimetry was performed, followed by OCT-A, SD-OCT, near infrared, and FAF image acquisition. There were no changes in equipment or procedures for the follow-up examinations of the participants imaged on more than one occasion. In all participants, the pupils were dilated with tropicamide 1% and phenylephrine 2.5% before retinal imaging. Macular SD-OCT scans averaged 100 A-scans/B-scan using the manufacturer's automatic retinal tracking software (Spectralis HRA + OCT; Heidelberg Engineering, Vista, CA, USA) to acquire 20-degree horizontal line scans through the center of the foveal avascular zone. Volume scans were acquired at 1-degree intervals to cover 20 degrees of the macula centered on the foveal avascular zone. The same system was used to obtain near infrared (central wavelength = 870 nm) and short-wavelength FAF images with a 488 nm light source for excitation in all 4 participants with macular atrophy.

### Optical Coherence Tomography-Angiography

High resolution vascular images were acquired using a swept-source OCT-A system (PLEX Elite 9000; Carl Zeiss Meditec Inc., Dublin, CA, USA) in all four participants with macular atrophy. Three-dimensional slab images were obtained by scanning a 6 mm × 6 mm area in a horizontal raster plan, as described previously.^27^ Choriocapillaris flow deficits within and around the PRL were quantified as previously described^28^^,^^29^ and summarized briefly as follows. The optical microangiopathy (OMAG) algorithm was used to identify blood flow in the scans. A semi-automated segmentation algorithm was used to identify the choriocapillaris slab, which extended from 4 µm to 20 µm below the outer border of Bruch's membrane (BM). An algorithm which comprised pre-processing and signal compensation was applied to the en face choriocapillaris slabs to compensate for choriocapillaris signal attenuation imposed by the structural changes in the RPE/BM complex. After setting the threshold at 1 standard deviation below the mean choriocapillaris flow of a database comprised of 20 healthy participants, the flow deficit percentages within and around the PRL were estimated in a 2-degree grid pattern throughout the macula, excluding areas of atrophy from the analysis.

### AOSLO Imaging

High-resolution images of cone photoreceptors in the central macular area of 5.7-degree diameter were obtained using confocal AOSLO. The AOSLO is a confocal scanning light ophthalmoscope that recorded 512 × 512 pixel videos of the retina over a 1.2 × 1.2-degree field at 30 hertz (Hz). The imaging wavelength was 840 nm. Blur-causing aberrations were corrected using adaptive optics. Wavefront measurement used a custom-built Shack Hartmann wavefront sensor operating with 910 nm light. Wavefront correction used a continuous membrane deformable mirror with 97 actuators (DM97; ALPAO, Montbonnot-Saint-Martin, France). Both the 840-nm light and the 910-nm light were drawn from a supercontinuum laser (SuperK EXTREME; NKT Photonics, Birkerod, Denmark) connected to a custom-built fiber coupling system. A sequence of videos spanning a 5 × 5 degree central region were collected with additional images extending 10 degrees along the horizontal midline and vertically from the foveal center until the superior retinal vascular arcade was imaged, to facilitate alignment with infrared fundus and FAF images. The digital videos were processed with the aid of custom image analysis software (MATLAB, The MathWorks, Inc., Natick, MA, USA) and montages were assembled were assembled using custom software (Automontage; https://github.com/BrainardLab/AOAutomontaging).^30^

### Cone Spacing Analysis

To assess the integrity of the cone mosaic in AOSLO images, we identified locations where cones mosaics were visible, selected the centers of a patch of contiguous cones within that region using semiautomated software,^31^ and then computed the average spacing of the cones using previously described methods. The cone spacing was compared against a previously reported database of 40 healthy eyes,^32^ and the spacing at each tested location was converted to a Z-score or number of standard deviations from the expectation for healthy eyes at that retinal location.

### AOSLO-Based PRL Determination and Fixational Analysis

The exact location of the PRL was determined using a feature of the AOSLO, whereby a fixation target is presented to the participants via direct projection within the raster scan. For this study, the fixation target was created by turning off the laser at a fixed location within the raster scan to present a black circle 0.12 degrees in diameter blinking at 6 Hz within the raster-scanned field.^33^ A fixation target delivered in this manner is encoded directly into the AOSLO video and the PRL used for fixation can be determined with absolute certainty.^21^ PRLs were determined for the 4 eccentric-fixating participants based on a 10-second video recording of the retina with the fixation target.

A more extensive quantification of fixation stability was obtained by extracting high-frequency eye motion traces from additional AOSLO videos that were used to generate the montage images.^34^ In these videos, the fixation target was not delivered via the raster as above, but a white cross (approximately 0.2 degrees in size) on a black background was presented with a second display that was viewed through a beamsplitter in the AOSLO system. During imaging, the participants were instructed to fixate as steadily as possible on the white cross over the course of each video. Ten eye motion traces were extracted from AOSLO videos from each participant. From the eye motion traces for the 4 macular atrophy participants, the BCEA containing 68% of fixation was calculated. The BCEA was then compared with those similarly collected from a group of four healthy, age-similar participants; four participants with RP due to pathogenic mutations in the *RHO* gene, which is expressed exclusively in rods; and eight participants with retinal degeneration associated with biallelic pathogenic mutations in the *USH2A* gene, which is expressed in rods and cones. Participants with *USH2A-*related RP were further divided into 2 groups based on visual acuity: BCVA < 20/30, or BCVA 20/30 to 20/50; clinical information is shown in the Table. BCEA between the cohorts were compared using a single-factor ANOVA followed by *t*-tests to determine the level of significance.

### Microperimetry

Fundus-guided microperimetry (Macular Integrity Assessment [MAIA]; CenterVue S.p.A, Padova, Italy) was used to measure macular sensitivities across a predefined set of locations in all participants. A dense custom grid extending every 1 degree from central fixation at least 4 degrees in the superior, inferior, nasal, and temporal directions was used to assess macular sensitivity around the PRL and in regions corresponding to high-resolution SD-OCT scans from the horizontal and vertical meridians from the PRL. A less dense, 10-2 full threshold strategy comprising a 68-stimuli grid covering the central 20 degrees of the retina was used to determine retinal sensitivities in participants with RP. Measurements were made in a semi-dark room using a Goldmann III stimulus presented for 200 msec. As per the MAIA user manual (www.icare-world.com/ifu/), the background luminance was 1.27 cd/m^2^ with a maximum stimulus luminance of 318.47 cd/m^2^ and the system used 25 Hz eye-tracking to guide the delivery of the test stimuli as well as to monitor fixation.

The MAIA offered independent measures of the PRL and fixation stability for each participant. During a MAIA measurement, participants are asked to hold fixation in the center of a 1-degree-diameter red ring. The instrument computes an initial PRL (*PRLi*) using the first 10 seconds of fixation, then uses all fixation positions over the course of the entire test to generate a final PRL (*PRLf*) as well as fixation indexes, which are quantified as the percentage of fixation points inside a circle of 2 and 4 degrees in diameter, respectively. Participants are classified as having stable fixation if greater than 75% of the fixation points are within 2 degrees, relatively unstable fixation if less than 75% of the fixation points are within 2 degrees and greater than 75% are within 4 degrees, and unstable fixation if less than 75% of fixation points are within 4 degrees, according to their product manual (www.icare-world.com/ifu/). MAIA also reports a BCEA that contains 63% of the fixation points during the scan, with the scan duration averaging 10 minutes in our participants.

### Combining the Data

All images and fundus-landmarked functional data from all structural and functional tests were aligned manually using vascular landmarks and were assembled into overlays in Adobe Illustrator (Adobe Systems, San Jose, CA, USA). Because the participants had central scotomas and the foveal structure (including the shape of the foveal pit) was compromised, the anatomic fovea for participants 40146, 40122, and 40184 was identified as the centroid of the hand drawn vessels bordering the edges of the foveal avascular zone on OCT-A images using public domain image processing software (ImageJ version 1.53e, https://imagej.net/ij/). The anatomic fovea for participant 30014 was identified as the PRL from the earlier visit where their central vision was intact.

## Results

### Structural Measures

Clinical information is summarized in the Table. Visual acuities in the study eyes ranged from 20/32 to 20/40 in the eyes with eccentric fixation. A heterozygous, pathogenic, mutation in the *BEST 1* gene (c. 920 C>T; p. Thr307Ile) was identified in participant 40146. In participant 30014, a pathogenic heterozygous single nucleotide change in the *ABCA4* gene (c.4457C>T, p.Pro1486Leu) was inherited in trans from a heterozygous insertion and deletion (c.672 703delinsN[6]; p.(Val225(?)fs*46)) in the *ABCA4* gene, as previously described. Next generation sequencing using a 181-gene retinal dystrophy panel revealed no disease-causing variants in participant 40184.

**Figure 1.**
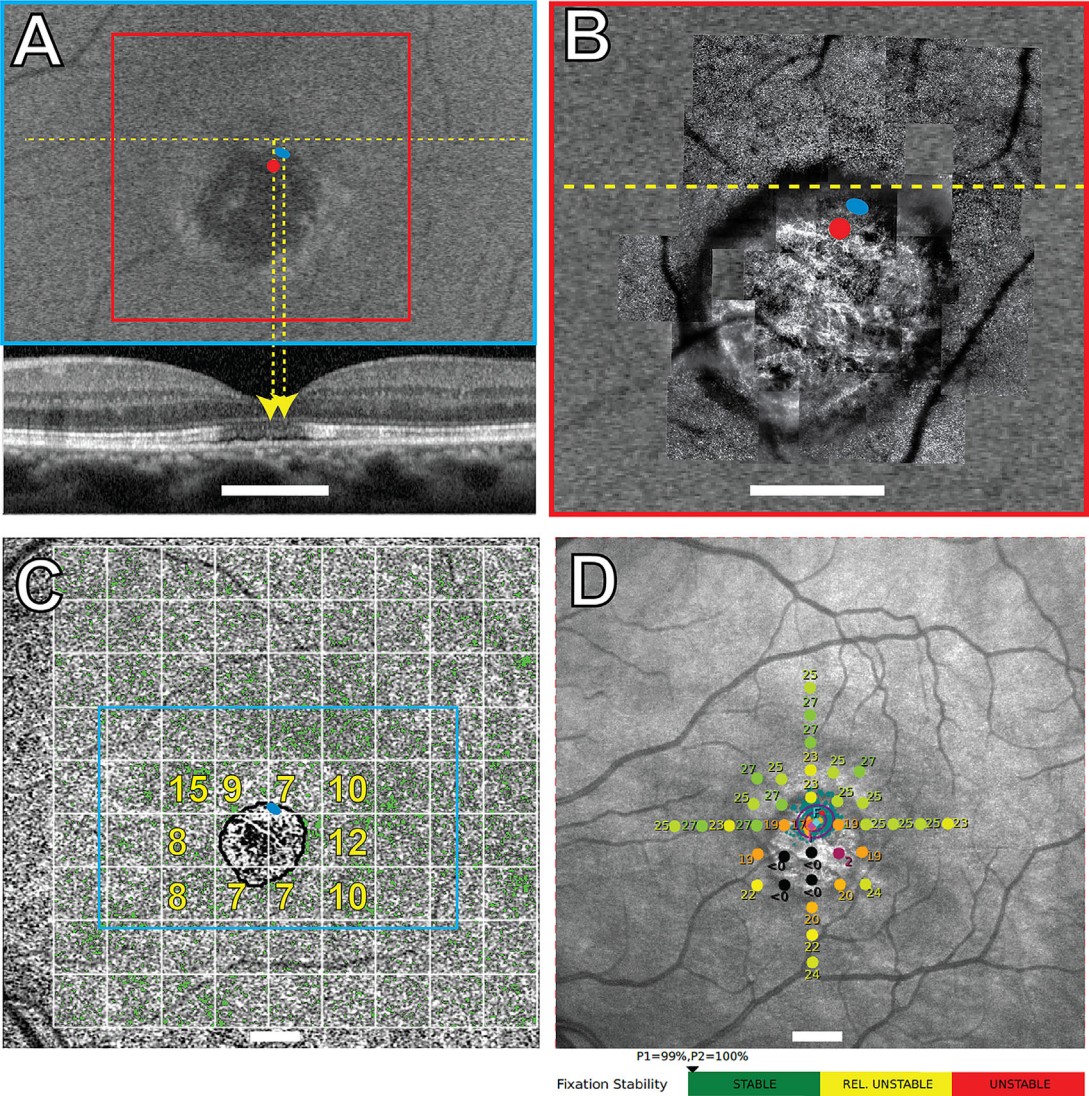
Participant 40146 left eye. Imaging and MAIA results. *Blue ellipses* in (**A, B,** **C**) indicate the AOSLO-determined PRL. *Red dots* in panels **A** and **B** indicate the locations of the anatomic fovea. (**A**) FAF image with the OCT B-scan shown below corresponding to the yellow dashed line. (**B**) AOSLO image corresponding to the region in the *red square* in panel **A**. (**C**) Green pixels show OCTA flow deficits with the cyan rectangle corresponding to the FAF image in panel **A**. Flow deficit percentages for each 2-degree area shown with a *white grid* are indicated in *yellow* text for selected areas around the atrophy. (**D**) MAIA sensitivity levels in decibels marked by *colored circles*, where worse sensitivity is *red* and normal is *green*; the *black* indicates that the brightest stimulus was not seen at that location. The location of the initial PRL, or *PRLi*, is indicated by the *magenta diamond* and the *magenta* letter “I.” The final PRL, or *PRLf*, is from the MAIA-determined fixation locations that are indicated by *small aqua pixel dots*, the corresponding BCEAs containing 63% and 95% of the fixation locations indicated by the *smaller* and *larger purple ellipses*, the *cyan diamond symbol*, and the *cyan* letter “F.” The MAIA fixational stability is classified as stable. Scale bar in all panels = 2 degrees.

**Figure 2.**
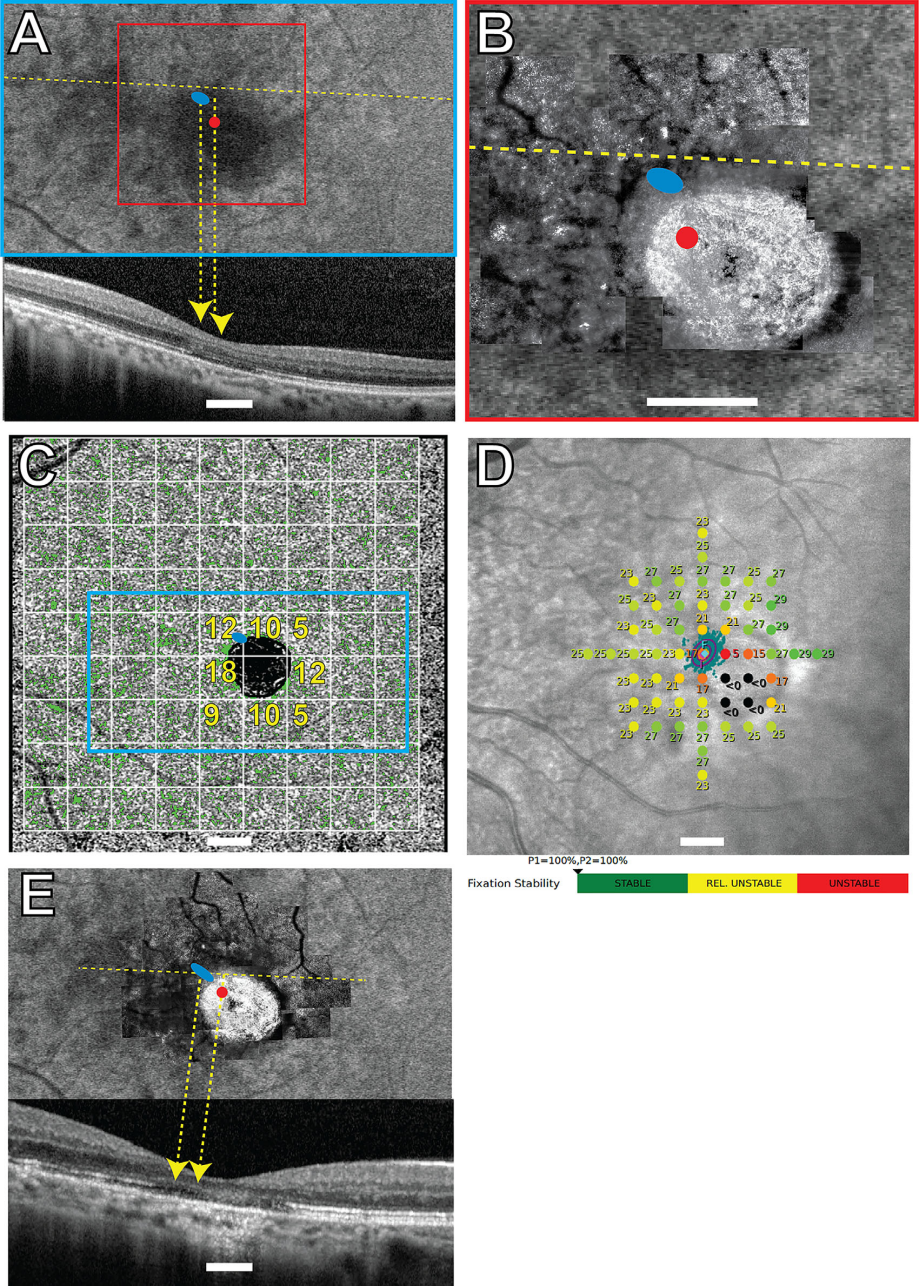
Subject 40122 left eye. Imaging and MAIA results. *Blue ellipses* in (**A, B, C,** **E**) indicate the AOSLO-determined PRL. *Red dots* in panels **A**, **B**, and **E** indicate the locations of the anatomic fovea. (**A**) FAF image with the OCT B-scan shown below corresponding to the *yellow dashed line*. (**B**) AOSLO image corresponding to the region in the *red square* in panel **A**. (**C**) Green pixels show OCTA flow deficits with the *cyan rectangle* corresponding to the FAF image in panel **A**. Flow deficit percentages for each 2-degree area shown with a *white grid* are indicated in *yellow text* for selected areas around the atrophy. (**D**) MAIA sensitivity levels in decibels marked by *colored circles*, where worse sensitivity is *red* and normal is *green*; the *black* indicates that the brightest stimulus was not seen at that location. The location of the initial PRL, or *PRLi*, is indicated by the *magenta diamond* and the *magenta* letter “I.” The final PRL, or *PRLf*, is from the MAIA-determined fixation locations that are indicated by *small aqua pixel dots*, the corresponding BCEAs containing 63% and 95% of the fixation locations indicated by the *smaller* and *larger purple ellipses*, the *cyan diamond symbol*, and the *cyan* letter “F.” The MAIA fixational stability is classified as stable. (**E**) Baseline imaging session from 1.5 years prior with the AOSLO superimposed on the FAF and the SD-OCT B-scan shown below. Scale bar in all panels = 2 degrees.

**Figure 3.**
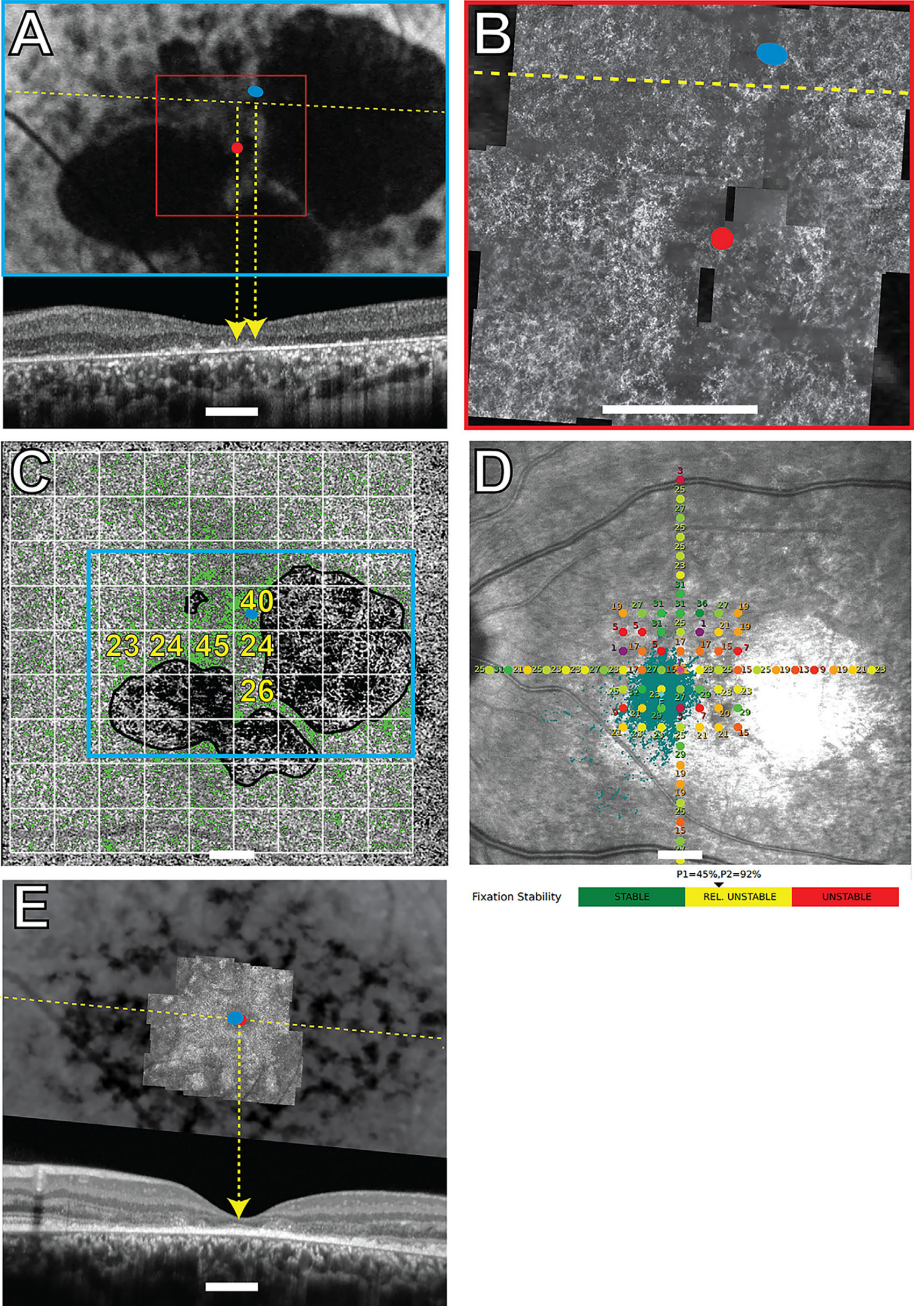
Subject 30014 left eye. Imaging and MAIA results. *Blue ellipses* in (**A, B, C,** **E**) indicate the AOSLO-determined PRL. *Red dots* in panels **A**, **B**, and **E** indicate the locations of the anatomic fovea. (**A**) FAF image with the OCT B-scan shown below corresponding to the *yellow dashed line*. (**B**) AOSLO image corresponding to the region in the *red square* in panel **A**. The *dark rectangular patches* are missing sections of the AOSLO montage. (**C**) *Green pixels* show OCTA flow deficits with the *cyan rectangle* corresponding to the FAF image in panel **A**. Flow deficit percentages for each 2-degree area shown with a *white grid* are indicated in *yellow text* for selected areas around the atrophy. (**D**) MAIA sensitivity levels in decibels marked by *colored circles*, where worse sensitivity is *red* and normal is *green*; the *black* indicates that the brightest stimulus was not seen at that location. MAIA-determined fixation locations are indicated by *small aqua pixels*. The BCEA is not shown due to unstable fixation. (**E**) Baseline imaging from almost 9 years prior with the AOSLO superimposed on the FAF and the SD-OCT B-scan shown below. At baseline, the subject still used their anatomic fovea for fixation. Scale bar in all panels = 2 degrees.

**Figure 4.**
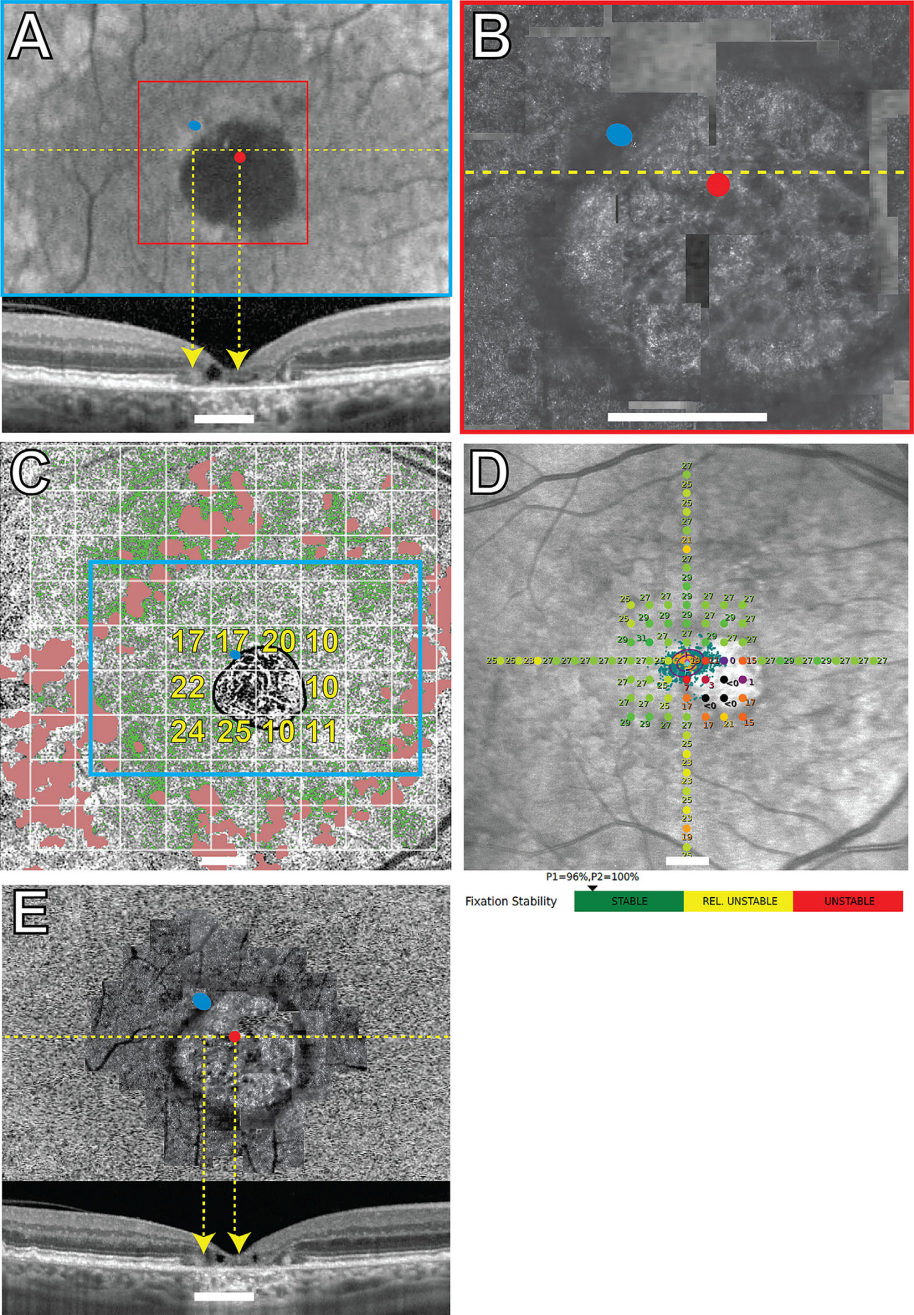
Subject 40184 right eye. Imaging and MAIA results. *Blue ellipses* in (**A, B, C,** **E**) indicate the AOSLO-determined PRL. *Red dots* in panels **A**, **B**, and **E** indicate the locations of the anatomic fovea. (**A**) FAF image with the OCT B-scan shown below corresponding to the *yellow dashed line*. (**B**) AOSLO image corresponding to the region in the *red square* in panel **A**. The *dark rectangular patches* are missing sections of the AOSLO montage. (**C**) *Green pixels* show OCTA flow deficits with the *cyan rectangle* corresponding to the FAF image in panel **A**. Flow deficit percentages for each 2-degree area shown with a *white grid* are indicated in *yellow text* for selected areas around the atrophy. *Pink patches* in participant 40184 indicate areas with drusen. (**D**) MAIA sensitivity levels in decibels marked by *colored circles*, where worse sensitivity is *red* and normal is *green*; the *black* indicates that the brightest stimulus was not seen at that location. The location of the initial PRL, or *PRLi*, is indicated by the *magenta diamond* and the *magenta* letter “I.” The final PRL, or *PRLf*, is from the MAIA-determined fixation locations that are indicated by *small aqua pixel dots*, the corresponding BCEAs containing 63% and 95% of the fixation locations indicated by the *smaller* and *larger purple ellipses*, the *cyan diamond symbol*, and the *cyan* letter “F.” The MAIA fixational stability is classified as stable. (**E**) Baseline imaging session from 3 months prior with the AOSLO superimposed on the FAF and the SD-OCT B-scan shown below. Scale bar in all panels = 2 degrees.

All the structural and functional data for participants 40146, 40122, 30014, and 40184 are shown in Figures 1 to 4, respectively. Participants 40122, 40184, and 30014 were imaged twice with 1.5 years, 3 months, and 9 years between imaging sessions, respectively. The overlays from the earlier visits are shown in Figures 2E, 3E, and 4E. The PRL was located superotemporal to the anatomic fovea in participants 40146, 40184, and 30014 in the most recent images, and superonasal to the anatomic fovea in participant 40122. These are best seen in Figures 1A–B, 2A–B, 3A–B, and 4A and 4B. SD-OCT B-scans showed the external limiting membrane band (ELM) was visible overlying hyporeflective, disrupted IS/OS junction and cone outer segment tip bands at the PRL in each study eye (Figs. 1A, 2E, 3A, 3E, 4A, 4E). BCEAs containing 63% of the fixational eye movement during MAIA microperimetry (depicted as the smaller of the two purple ellipses in Figs. 1D, 2D, 4D) were larger than, and did not exactly match, the location of the BCEAs determined by AOSLO (blue ellipses in Figs. 1A–C, 2A–C, 2E, 3A–C, 3E, 4A–C, 4E). MAIA estimates of BCEA are not shown for participant 30014 due to unstable fixation when tested with MAIA. The PRL was located between 0.43 and 2.45 degrees from the anatomic fovea in all four eyes in the most recent images. Participants 40122 and 40184 did not change the location of their PRL between visits (see Figs. 2E, 4E, 1.5 years, and 3 months earlier, respectively). The PRL for participant 30014 coincided with the anatomic fovea at the first visit (see Fig. 3E), but 9 years later it had shifted 2.45 degrees superiorly (see Fig. 3A). Unambiguous cones were not visible at the PRL in confocal AOSLO images in the four primary participants, even though more normal-appearing cones were located elsewhere in the images at greater eccentricities than the PRL. Supplementary Figures S1, S2, and S3 show higher magnification AOSLO images of participants 40146, 40122, and 40184. Insets show regions chosen as close as possible to the PRL or to the scotoma with contiguous mosaics of cones that were selected for spacing analysis. The Z-scores indicated normal spacing at all tested locations. Unambiguous cones were not visible anywhere in participant 30014.

RPE structure and choriocapillaris perfusion were also abnormal at the PRL. FAF showed reduced and/or heterogeneous autofluorescence at and around the PRL in all four eyes (see Figs. 1A, 2A, 3A, 4A). SS-OCTA images of choriocapillaris flow deficit in the macular area in healthy eyes has been reported as 10.31 ± 3.66%.^28^ Choriocapillaris flow deficit within the PRL in the most recent images was normal in participant 40146 (7%) and participant 40122 (12%) but increased in participant 30014 (40%) and participant 40184 (17%; green pixels, see Figs. 1C, 2C, 3C, 4C). The PRL in participant 40184 was located at a region with increased flow deficit even though there were regions at similar eccentricities with more normal choriocapillaris flow (e.g. approximately 10% at 3 locations at the nasal edge of the macular atrophy in Fig. 4C).

### Functional Measures

Mean retinal sensitivities in normal healthy eyes using MAIA have been previously reported as 29.26 decibels (dB), 28.19 dB, and 27.31 dB, respectively, at 0 to 2, 2 to 6, and 6 to 10 degrees from fixation.^35^ Mean retinal sensitivities were abnormal at several locations throughout the macula in participants 40146, 40122, and 40184, and were severely reduced at the PRL at 17 dB, 17 dB, and 19 dB, respectively (see Figs. 1D, 2D, 4D). Participants 40146, 40122, and 40184 maintained fixation within 2 degrees of the target between 96% and 100% of the time (reported just below Figs. 1D, 2D, 4D). Participant 30014 did not appropriately attend to the task during the most recent MAIA session with relatively unstable fixation (MAIA BCEA = 5.3 degrees^2^) and 8 of 12 targets to the optic nerve marked as seen, so retinal sensitivities were not reliable enough to report for this participant.

### Fixational Measures

**Figure 5.**
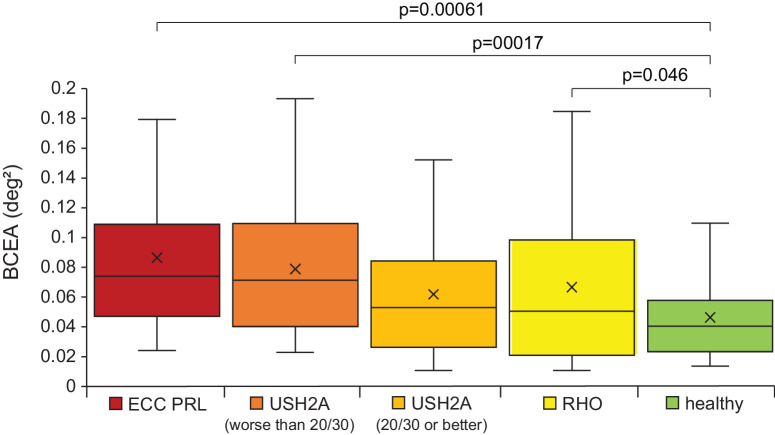
AOSLO BCEA measurements in degrees^2^ for participants with macular atrophy and eccentric PRL (ECC PRL, *red*), participants with *USH2A*- related retinitis pigmentosa and visual acuity worse than 20/30 (*dark orange*) and 20/30 or better (*orange*), participants with *RHO* -related retinitis pigmentosa (*yellow*), and healthy participants (*green*). For these box and whisker plots, the *top* and *bottom edge* of each box represents the 75th and 25th percentiles of the distribution, respectively, the median is represented by the *horizontal line* inside the box, the “x” symbol indicates the mean value. The upper and lower termini of the whiskers indicate the 95th and 5th percentiles of the distribution, respectively. Each category includes data from four participants (see the Table) and each participant has 10 BCEA values computed from 10 different videos. All *P* values less than 0.05 are indicated on the plot.

BCEA measured by AOSLO was analyzed in 10 eye motion traces from each of the participants in each of these 5 groups: participants with eccentric fixation, participants with *USH2A-*related retinal degeneration and visual acuity 20/50 to 20/30, participants with *USH2A-*related retinal degeneration and visual acuity better than 20/30, participants with *RHO*-related RP, and healthy participants (Fig. 5). There were no participants with *RHO-*related RP and visual acuity worse than 20/30 (see the Table). There was no significant difference in BCEA in degrees^2^ between the participants with eccentric fixation and the participants with Usher syndrome or RP regardless of visual acuity (*P* = 0.052 for comparison with *USH2A* with BCVA equal to or better than 20/25, *P* = 0.55 for comparison with participants with *USH2A* with BCVA equal to or worse than 20/30, and *P* = 0.15 for comparison with participants with *RHO* mutations). There was a significant difference in BCEA between participants with eccentric fixation and healthy participants (*P* = 0.00061), between participants with *USH2A-*related RP and visual acuity worse than 20/30 and healthy participants (*P* = 0.00017), and between *RHO-*related RP and healthy participants (*P* = 0.046). There was no significant difference between any groups in age, and when combining all groups, neither age nor visual acuity were significantly correlated with BCEA. Retinal sensitivity at the PRL measured in MAIA for all groups except the healthy controls was also not significantly correlated with BCEA.

**Figure 6.**
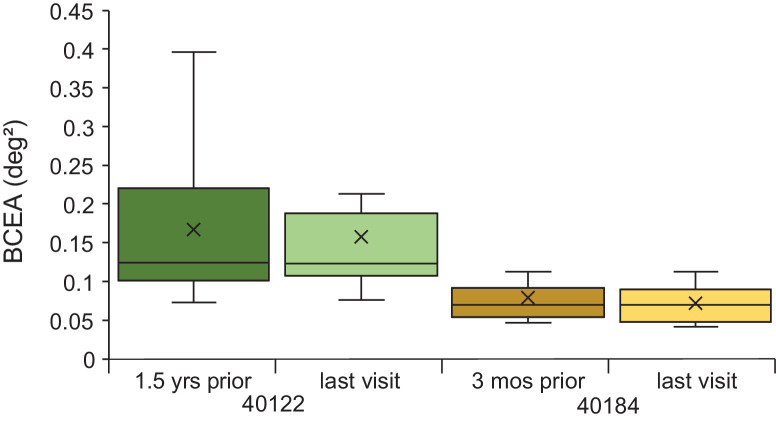
AOSLO bivariate contour ellipse area measurements for participants 40122 with rubella measured 1.5 years apart and 40184 with cuticular drusen measured 3 months apart. Each visit included BCEAs computed from 10 AOSLO videos. The box and whisker plot definitions are the same as described in Figure 5.

BCEA for participants 40146, 30014, and 40184 with eccentric fixation were not significantly different from one another, but all 3 had a significantly smaller BCEA than participant 40122 with congenital rubella. Participants 40122 and 40184 did not change BCEA significantly when measured over a 1.5-year or 3-month period, as shown in Figure 6.

## Discussion

### Location of PRL

Using a multi-modal, high-resolution imaging approach, the present study of the PRL in participants with macular atrophy from four different conditions revealed that proximity of the PRL to the anatomic fovea is more important than integrity of retinal structure or function at the PRL. In four out of four cases of macular atrophy due to different causes, participants used a retinal region with reduced cone reflectivity, abnormal outer retinal structures on SD-OCT, heterogeneous FAF, reduced choriocapillaris perfusion, and lower sensitivity compared with regions at greater eccentricities.

Prior reports have shown wide variation in the location of the PRL among participants with macular diseases.^3^^,^^4^^,^^6^^–^^9^^,^^11^^,^^14^ Cheung and Legge^1^ hypothesized that the location of the PRL could be function-driven, performance-driven, or retinotopic-driven, defined as follows. A *function-driven* PRL describes placement of the PRL in the area of retina outside of the fovea that is most appropriate for a specific visual activity; for example, a superiorly located PRL may improve visual function for lower visual field tasks, such as reading or walking, by moving the scotoma into the upper visual field. PRLs have also been previously shown to shift depending on the lighting conditions in participants with central scotomas by an average of 4.6 degrees, and by as much as 12 degrees, with a general shift to more superior retinal PRLs in low lighting conditions, like those used in this study.^36^ A *performance–driven* PRL describes placement of the PRL at the area of the retina that gives the best visual acuity. A *retinotopic-driven* PRL describes a PRL located close to the edge of the macular atrophy that could best serve visual function based on retinotopic proximity to the anatomic fovea. Note that these criteria are rarely mutually exclusive. In the present study, the PRL was located superior to the anatomic fovea, and adjacent to the edge of the macular atrophy in all four eyes (0.43–2.45 degrees from the fovea). The current manuscript supports the *function-driven* and *retinotopic-driven* hypotheses, as in our small sample, the location of the PRL was located at the region superior and closest to the anatomic fovea with measurable visual function. We cannot conclude if the *performance-driven* criteria is met since, although the PRL in our participants was located at a region with lower retinal sensitivity, we do not know to what extent the PRL locations compromise visual acuity.^37^

### RPE and Choriocapillaris Abnormalities at the PRL

A homogenous distribution of fundus autofluorescence does not appear to be a strong criterion dictating the PRL placement. The PRL was located in regions with heterogenous autofluorescence in all four eyes (see Figs. 1A–4A). In participants 40122, 40146, and 40184, more normal-appearing FAF findings were present at greater eccentricities than the PRL, with participant 30014 showing more widespread FAF abnormalities. FAF abnormalities indicate RPE abnormalities, which may compromise wave-guiding cone outer segments and sensitivities at the PRL.

Choriocapillaris flow does not appear to be a strong criterion dictating the placement of the PRL. Choriocapillaris flow was reduced at the PRL in participant 30014 with Stargardt disease and in participant 40184 with macular atrophy associated with cuticular drusen but was within normal limits in participants 40146 with Best disease and 40122 with congenital rubella. In all participants, choriocapillaris flow was closer to normal at increased eccentricities. Notably, in participant 40184, flow deficits that were closer to normal could be found at similar eccentricities from the anatomic fovea as the PRL. The differences in choriocapillaris flow deficits observed among the four participants in the current study could reflect the variation in the pathogenic mechanisms and rate of progression of the four different diseases. The increased flow deficit in participants 30014 and 40184 suggest that choriocapillaris perfusion is more abnormal in Stargardt disease and macular degeneration associated with cuticular drusen than in congenital rubella (40122) and Best disease (40146). Prior studies have demonstrated impaired choriocapillaris blood flow within, around, and beyond the margins of GA in non-neovascular AMD,^38^^–^^41^ as we also observed in 40184 with macular degeneration and cuticular drusen, and also in participant 30014 with Stargardt disease. A case report of macular atrophy studied with OCT-A showed a vascular network surrounding macular excavation,^42^ but flow deficit has not been previously reported, and choriocapillaris flow deficit has not been reported in participants with macular atrophy from Best disease, to our knowledge.

### Fixation Differences With an Eccentric PRL

This study found a significant difference in BCEA between eccentric fixators and healthy participants, which is in line with previous studies comparing eccentric fixators to healthy age-matched participants.^12^ The BCEA increase may not solely result from eccentric fixation in our participants, as the BCEA in our eccentric fixation cohort was not significantly different from participants with RP or Usher syndrome type 2 that had central fixation. The lack of difference in BCEA between eccentric and central fixators indicates that fixational stability may depend more on the number and health of the cones at the PRL than the PRL location. The participants with *USH2A*-related retinal degeneration and visual acuity worse than 20/30 were significantly different from normal, indicating that fixational instability may be related to visual acuity.

Previous studies have found an increase in fixational instability with increasing age and worsening visual acuity^43^; however, no such correlation was found in this study, perhaps due to lack of power in the current study.

### Limitations

The present study included four participants (4 eyes) with different diseases and who all had eccentric PRLs close to the anatomic fovea. This means that our results have to be interpreted cautiously in the following ways. First, the results may inform, but should not be extrapolated to predict, structure and function of the PRL at greater eccentricities. Second, the small number of study eyes means that we cannot rule out different behavior in individuals with the same retinal disease. Third, although every subject had a PRL that was located closer to the fovea than regions with apparently more normal retina, RPE, choroidal structure, and visual function, this finding may not extend to macular pathologies that are not included in the limited range of conditions studied. Fourth, we chose the centroid of the foveal avascular zone (FAZ) as the anatomic foveal center to compute the PRL eccentricities for three of the four participants. Although this location is known to not exactly correspond to the normal PRL or to the location of peak cone density,^44^ it was our only option due to the absence of other functional or visible anatomic landmarks. Variability in the identification of the anatomic foveal location will consequently result in variability in the computation of small PRL offsets reported here.

The MAIA results must be also interpreted with some caution: Figures 1D, 2D, 3D, and 4D show many instances where MAIA fundus-guided microperimetry indicate measurable sensitivity values in regions of apparent RPE and retinal atrophy shown by SD-OCT, FAF, and AOSLO. This suggests potentially imprecise fundus localization of the visual stimulus using fundus-guided microperimetry.

The PRLs reported here were measured under monocular viewing conditions at a single visit. It is possible that different PRLs may be chosen when tested under binocular conditions where the better eye drives the location of the PRL.

Finally, BCEA measured using AOSLO should not be directly compared with BCEA from the MAIA, for several reasons. First, the fixation targets were different; the AOSLO used a small, blinking black dot in a red field, whereas the MAIA used a red ring 1 degree in diameter. Second, the AOSLO measured fixational eye movements over 5-second segments at 960 Hz, while the participants were instructed to fixate, whereas the MAIA BCEA was computed based on eye movements measured at 25 Hz over a much longer, continuous 10-minute period.

## Conclusions

The PRL in individuals with central macular atrophy affecting the anatomic fovea is in regions that are structurally and functionally compromised in almost every measurable way: cone reflectivity/waveguiding, choriocapillaris perfusion, RPE health, and retinal sensitivity are all worse at the PRL than at more peripheral locations. The two clear priorities determining the location of an eccentric PRL are that the PRL is generally positioned superior (nasal or temporal) to the anatomic fovea and close to the anatomic fovea. Whether other PRLs might provide improved visual function or fixation stability has not been assessed; evaluating different PRLs may prove difficult as participants have likely adapted to this new location.

## Supplementary Material

Supplement 1
