# Supplementary material for: High Resolution Imaging and Fixation Analysis of Eccentric Preferred Retinal Loci in Macular Diseases
Source: Invest Ophthalmol Vis Sci. 2025 May 8;66(5):18. doi: 10.1167/iovs.66.5.18 (PMC12068526; doi:10.1167/iovs.66.5.18)
Supplement: Supplement 1 [file iovs-66-5-18_s001.pdf]

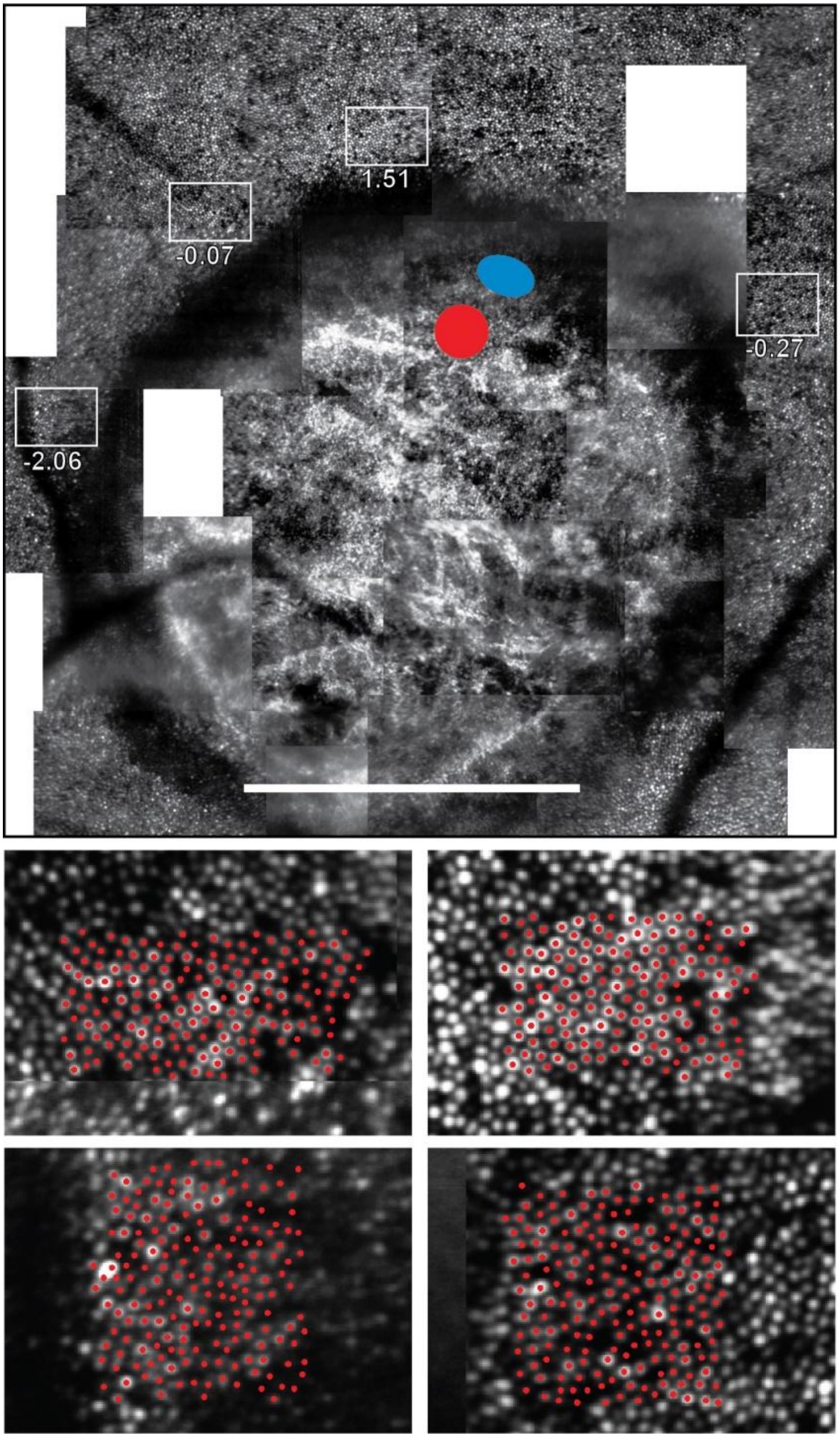
1.51
-0.07
-0.27
-2.06

Figure S1: Participant 40146 AOSLO image. Upper panel: AOSLO image with the anatomic fovea (red circle) and PRL (blue ellipse) indicated. The rectangles show the location of the insets (lower panels) where mosaics of cone were identified, and cone spacing Z-scores were computed. The Z-scores are adjacent to each rectangle. Z-scores at three of the four locations are within +/- 2 standard deviations of the distribution of normal cone spacing for their respective locations relative to the fovea. Scale bar is 2 degrees.

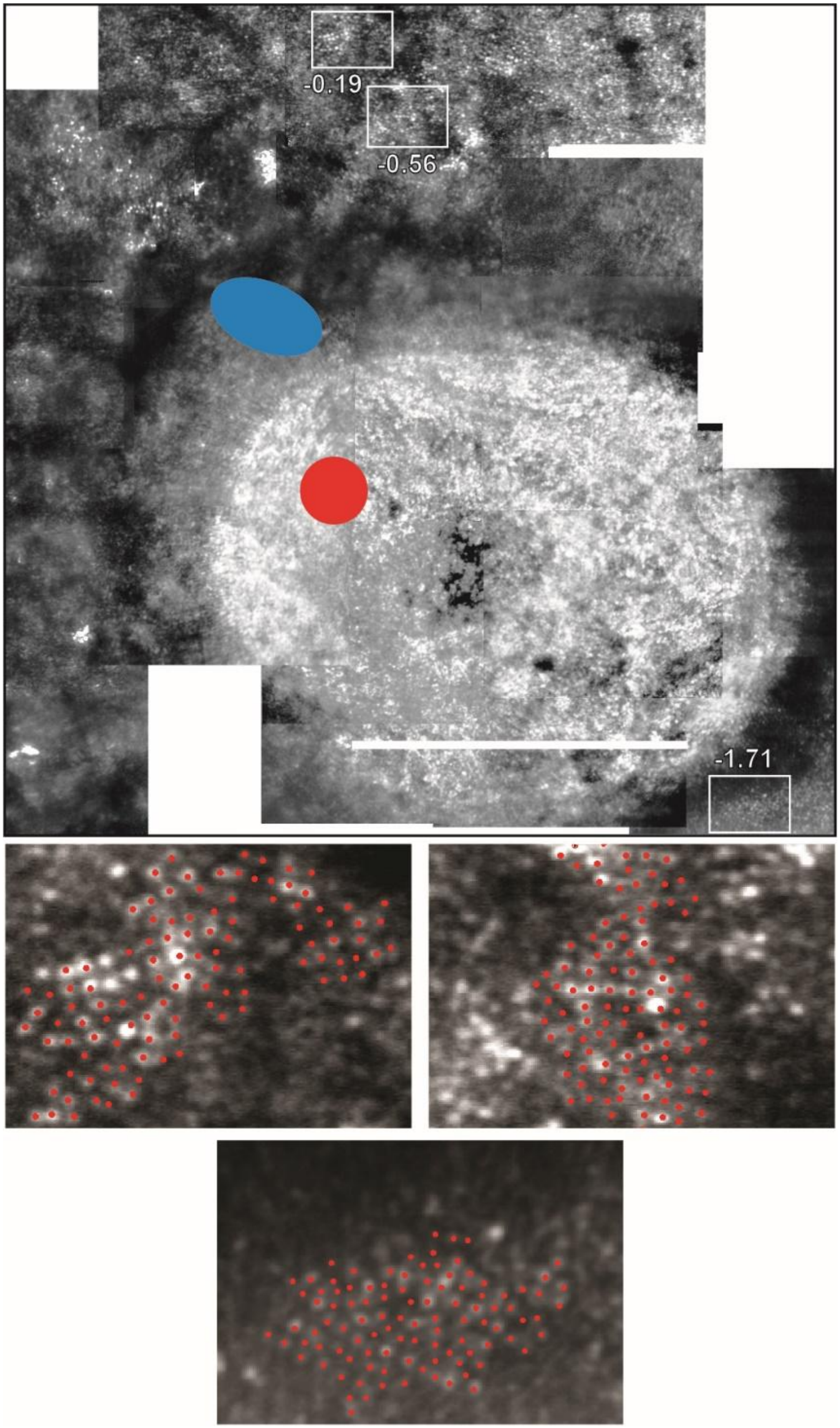

-0.19
-0.56
-1.71

Figure S2: Participant 40122 AOSLO image. Upper panel: AOSLO image with the anatomic fovea (red circle) and PRL (blue ellipse) indicated. The rectangles show the location of the insets (lower panels) where mosaics of cone were identified, and cone spacing Z-scores were computed. The Z-scores are adjacent to each rectangle. Z-scores at all three locations are within +/- 2 standard deviations of the distribution of normal cone spacing for their respective locations relative to the fovea. Scale bar is 2 degrees.

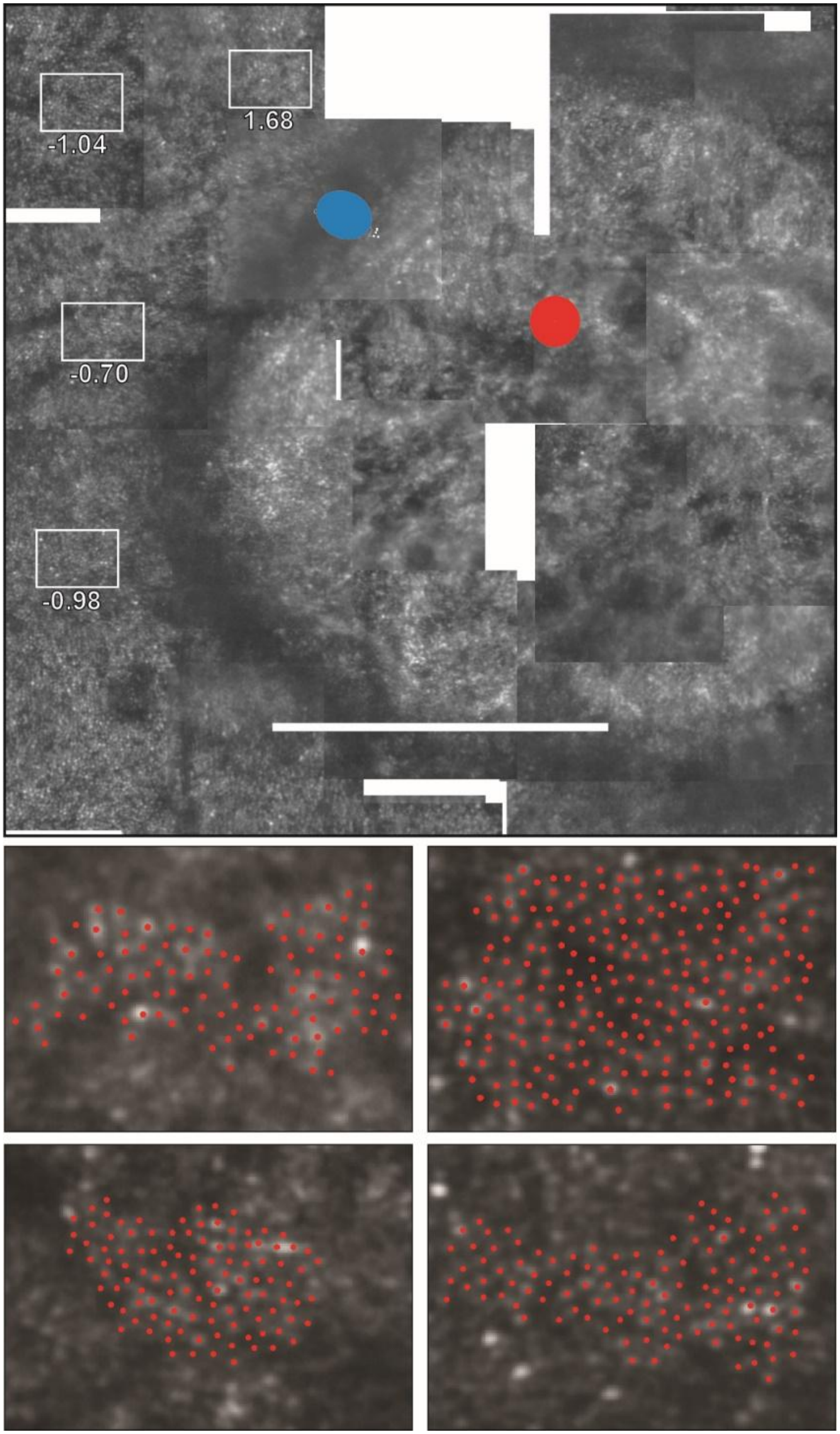

-1.04
1.68
-0.70
-0.98

Figure S3: Participant 40184 AOSLO image. Upper panel: AOSLO image with the anatomic fovea (red circle) and PRL (blue ellipse) indicated. The rectangles show the location of the insets (lower panels) where mosaics of cone were identified, and cone spacing Z-scores were computed. The Z-scores are listed adjacent to each rectangle. Z-scores at all four locations are within +/- 2 standard deviations of the distribution of normal cone spacing for their respective locations relative to the fovea. Scale bar is 2 degrees.
